# 

**Published:** 1869-03-01

**Authors:** 


### *CONTENTS.*

TRANSLATIONS:

The Therapeutic Employment of the Fumes of Opium.... 129

Reflex Actions Determined by Constant and Continuous Electric Currents. 133

ORIGINAL COMMUNICATIONS:

Case.—Amputation at the Shoulder Joint.—Recovery. By Hiram Wanzer, M. D., Chicago.... 134

Cook County Hospital, Chicago, Ills.—Rupture of Urethra—Cystotomy—Cured. Service of Dr. Edwin Powell. B. C. Miller, House Surgeon.... 137

Super-Oxygenation as a Thereapeutic Measure, with Notes of Cases Treated. By S. S. Wallihan, M. D., Chicago. (Concluded).... 139

PROCEEDINGS OF SOCIETIES:

Rush Medical College—Alumni Meeting.... 152

American Medical Association.... 154

FOREIGN ITEMS: 155

EDITORIAL:

The Last Number—Medical Legislation in Illinois—Svapnia and Sweet Quinine.... 156

BOOKS RECEIVED:

On the Dynamics, Principles and Philosophy of Organic Life, etc. By Z. C. McElroy, M. D, Zanesville, Ohio.... 157

A Hand Book of Uterine Therapeutics, and of Diseases of Women. By Edward John Tilt, M. D.,.... 158

The Health-Lift. Its Theory and Practice.... 158

Braithwaite's Retrospect of Practical Medicine and Surgery—Part LVIII.. 158

Archives of Ophthalmology and Otology. Prof. H. Knapp, M. D., and Prof. S. Moos, M. D.... 158

ITEMS:

Difficult and Tedious Labor—The Craig Microscope—The Summer Course, etc., at Jefferson Medical College—Lithograph—Special Notice to Snbscribers.... 159

Rush Medical College—Spring Announcement.... 160

